# Spontaneous uterine rupture with primary epigastric pain in the second trimester: a case report on diagnostic challenges and multidisciplinary management

**DOI:** 10.3389/fmed.2026.1805420

**Published:** 2026-04-09

**Authors:** Haiyan Wang, Na Meng, Hong Wang, Yanling Dong, Yuping Jiang, Xinjiang Li, Yingping Tian

**Affiliations:** 1 Department of Anesthesiology, The Second Hospital of Hebei Medical University, Shijiazhuang, Hebei, China; 2 Department of Emergency, The Second Hospital of Hebei Medical University, Shijiazhuang, Hebei, China; 3 Department of Anesthesiology, Shijiazhuang Obstetrics and Gynecology Hospital, Shijiazhuang, Hebei, China; 4 Department of Obstetrics, The Second Hospital of Hebei Medical University, Shijiazhuang, Hebei, China; 5 Department of Emergency Surgery, The Second Hospital of Hebei Medical University, Shijiazhuang, Hebei, China

**Keywords:** diagnostic challenges, epigastric pain, in the second trimester, multidisciplinary management, spontaneous uterine rupture

## Abstract

**Background:**

Uterine rupture during the second trimester is an exceptionally rare but life-threatening obstetric complication, presenting significant diagnostic and therapeutic challenges.

**Case summary:**

A 33-year-old woman, gravida 4, para 1, at 22 weeks and 3 days of gestation, was admitted due to a shortened cervical length. Four hours after admission, the patient developed acute, severe epigastric pain radiating to the back and shoulders. Her condition deteriorated with persistent hemoglobin decline. Abdominal and contrast-enhanced CT scans revealed perihepatic, perisplenic, and perienteric fluid accumulation, strongly suggesting intra-abdominal hemorrhage. Diagnostic paracentesis failed to yield non-coagulating blood. Following multidisciplinary consultation involving obstetrics, surgery, radiology, and anesthesiology, exploratory laparoscopy was performed to identify the bleeding site after obtaining informed consent, with conversion to laparotomy if necessary. Intraoperative findings revealed a full-thickness uterine rupture (4 cm × 1.5 cm) on the anterior wall. After further discussion with the family, cesarean delivery was performed, and the neonate was transferred to NICU. Postoperatively, the patient was admitted to ICU and received antibiotic therapy, fluid resuscitation, and thromboprophylaxis, achieving stable condition for discharge after 5 days. At the three-month postoperative follow-up, the patient recovered well.

**Conclusion:**

This case highlights the importance of multidisciplinary collaboration and timely laparoscopic exploration for acute abdominal pain during the second trimester, particularly in patients with intra-abdominal hemorrhage. Early identification and prompt surgical intervention remain critical for improving outcomes.

## 1 Introduction

Uterine rupture, defined as a full-thickness disruption of the uterine wall during pregnancy or labor, is a rare but life-threatening obstetric emergency, occurring in approximately 0.18% of pregnancies (1). Rupture during the mid-trimester is particularly uncommon and presents substantial diagnostic difficulties, especially when clinical manifestations resemble gastrointestinal pathology. Diagnostic imaging has inherent limitations: ultrasonography may fail to detect small uterine defects, while computed tomography (CT), although highly sensitive for identifying hemoperitoneum, frequently does not visualize the actual site of uterine disruption. We report a case of mid-trimester uterine rupture presenting predominantly with epigastric pain, underscoring the necessity for heightened clinical suspicion, effective multidisciplinary coordination, efficient vertical referral from specialized to general hospitals, and prompt surgical assessment.

## 2 Case presentation

### 2.1 History and physical examination

A 33-year-old woman, gravida 4, para 1, at 22 weeks and 3 days of gestation, was admitted due to a shortened cervical length (1.0 cm) identified on routine transvaginal ultrasound. Her obstetric history included two first-trimester miscarriages managed by dilation and curettage, as well as prior high-intensity focused ultrasound (HIFU) ablation of an anterior uterine fibroid in 2022. She had a diagnosis of uncontrolled gestational diabetes mellitus.

The vital signs at admission were: P: 98 beats/min, R: 20 breaths/min, BP: 117/82 mmHg. No tenderness was noted in the uterine body, and no uterine contractions were palpable. Fetal heart rate was140 beats/min.

Four hours after admission, the patient developed acute, severe epigastric pain radiating to the back and shoulders. Vital signs remained stable (temperature: 36.8 °C, pulse: 86 bpm, respiratory rate: 23 bpm, blood pressure: 128/76 mmHg, SpO₂: 100%). Physical examination revealed pallor, tenderness in the right upper quadrant that worsened with movement, a soft uterus with irregular and decreased contractions, and no evidence of vaginal bleeding or amniotic fluid leakage.

### 2.2 Investigations

**Figure 1:**
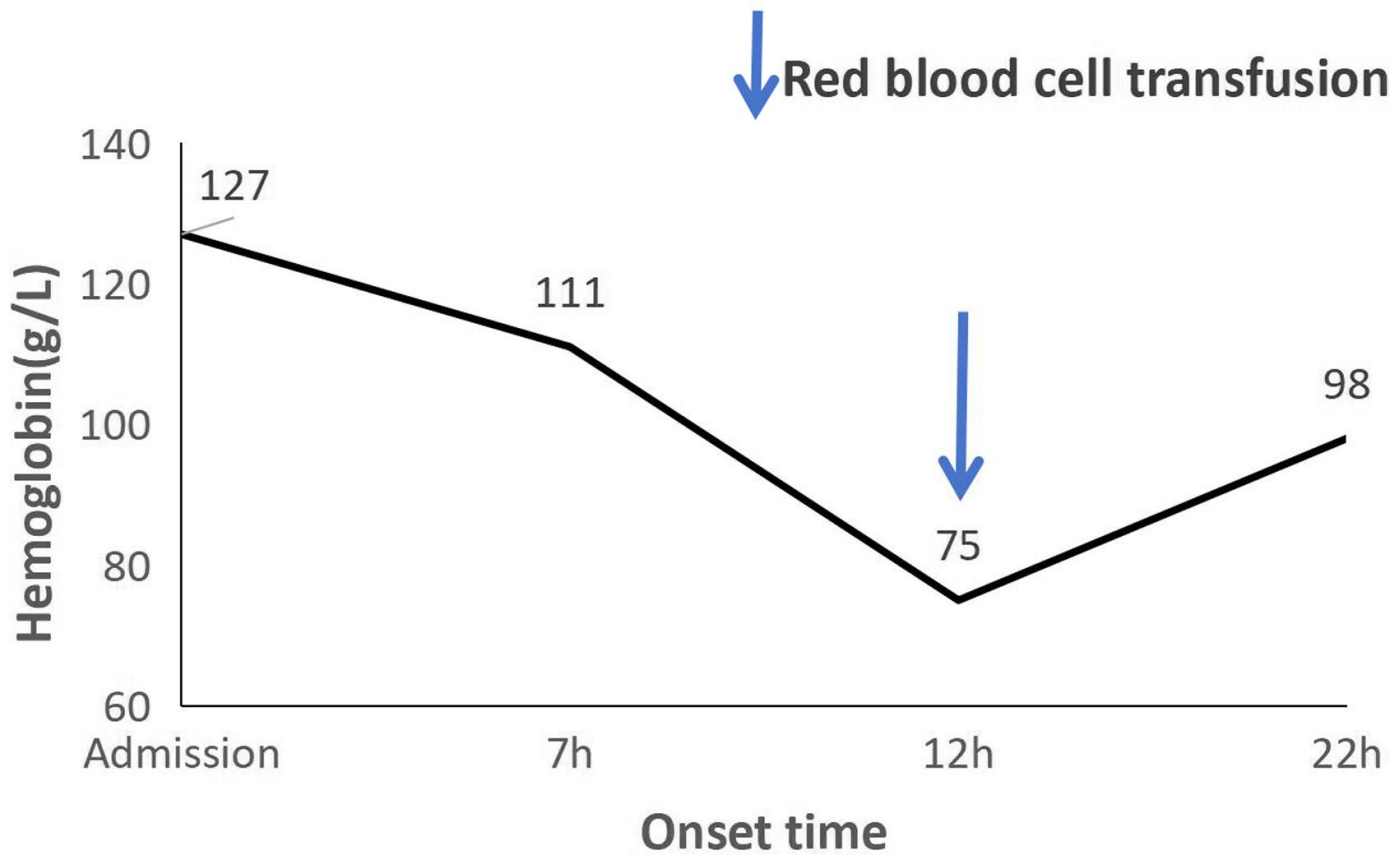
Hemoglobin trend after onset of disease: hemoglobin levels (g/L). Line graph depicting serial hemoglobin concentrations. Blue solid line: four unit red blood cell transfusion. Time axis: onset time. References: hemoglobin 110–150 g/L.

**Figure 2:**
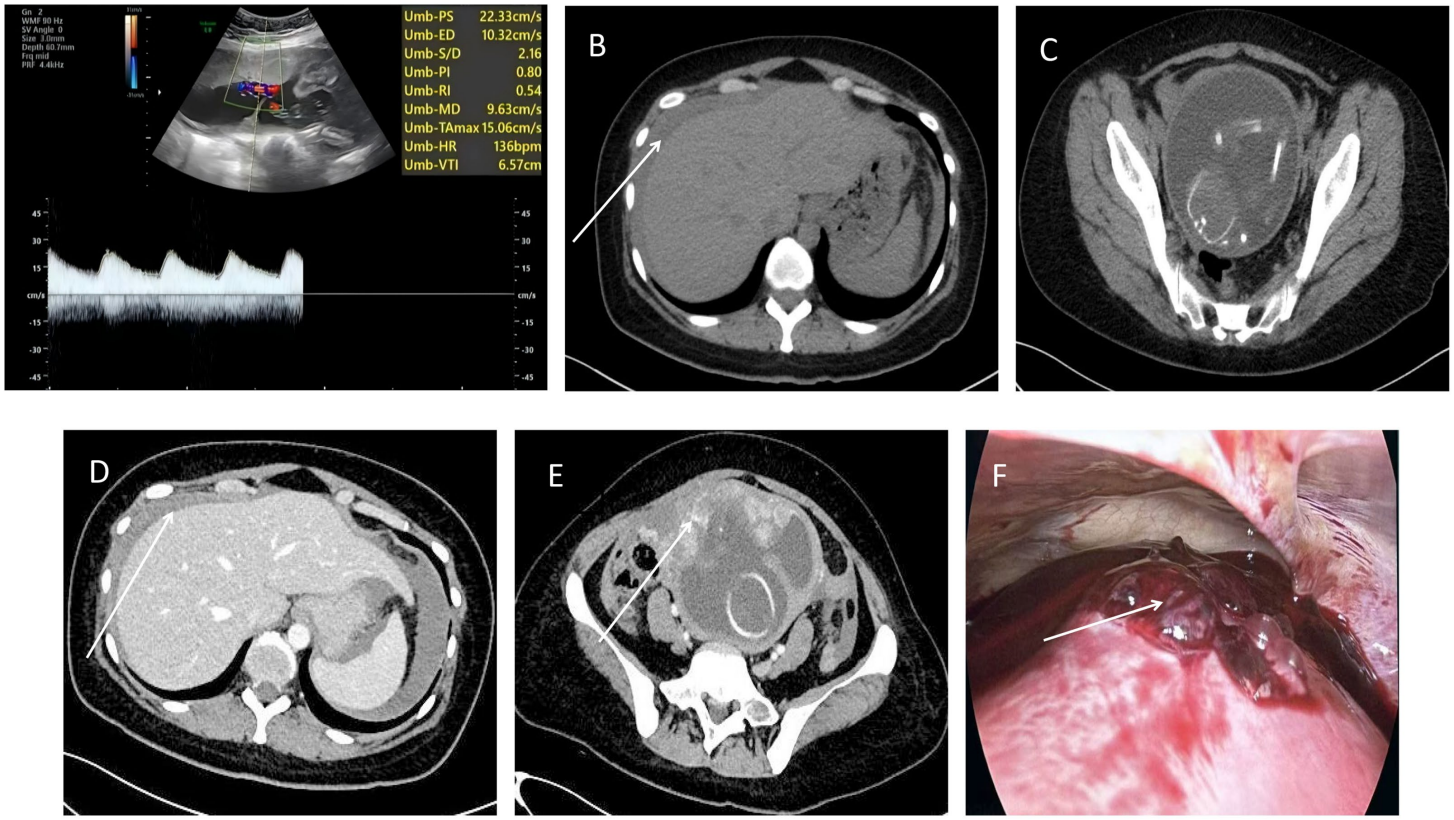
**(A)** Ultrasound indicates a single live fetus in intrauterine pregnancy. **(B)** A slightly high-density shadow can be seen around the liver and spleen, with a CT value of about 37–62 HU, which is considered as blood or fluid accumulation. **(C)** CT revealed the muscle layer and serosal layer are continuous. **(D)** There were many high-density fluid shadows in the perihepatic, perisplenic, bilateral paracolic sulci, mesenteric space, and pelvic cavity, suggesting massive hematoma or fluid accumulation in the abdominal and pelvic cavity. **(E)** The anterior wall of the uterus was slightly less clear. **(F)** Laparoscopy revealed a 4 cm × 1.5 cm rupture of the whole layer in the anterior wall of the uterus, 2 cm below the root of the round ligament (the abnormal region was indicated by the white arrow).

Laboratory assessment showed serial hemoglobin dropping from 127 g/L at admission to 111 g/L 7 h after symptom onset, and elevated D-dimer levels (600 μg/L), while cardiac enzymes, troponin, and serum amylase were within normal ranges (Figure 1). Fetal ultrasound revealed a single live fetus with a fetal heart rate of 136 beats per minute (Figure 2A). Electrocardiography was unremarkable. Non-contrast abdominal CT revealed pneumatosis intestinalis, small air-fluid levels, and hyperdense fluid collections in the perihepatic and perisplenic regions, suggestive of hemoperitoneum (Figures 2B,C). The uterine wall appeared intact. Subsequent contrast-enhanced CT confirmed a large volume of free intraperitoneal fluid without visualization of a definite source (Figures 2D,E). Diagnostic peritoneal aspiration did not yield non-coagulating blood.

### 2.3 Multidisciplinary consultation and surgical procedures

The clinical scenario was critical, necessitating an urgent multidisciplinary consultation involving obstetrics, general surgery, and radiology. The multidisciplinary team reached a consensus as follows: Radiologist: CT and contrast-enhanced CT findings were highly suggestive of an intra-abdominal hematoma, predominantly localized around the liver, spleen, and interloop spaces, with no overt signs of visceral organ injury. Surgeon: The patient presented with acute mid-upper abdominal pain radiating to the back as the primary clinical manifestation. Given the progressive decline in hemoglobin levels, hemorrhagic pathology was strongly suspected. Laparoscopic exploration was recommended to exclude gastrointestinal perforation and obstetric-related hemorrhagic conditions. Emergency laparoscopic exploration is required to identify the bleeding site, with laparotomy for hemostasis if necessary. Obstetrician: Abdominal and contrast-enhanced CT imaging revealed no definitive evidence of uterine rupture. However, considering the rarity of spontaneous uterine rupture during mid-gestation, diagnostic laparoscopy was advised to confirm or exclude this diagnosis.

Intraoperative laparoscopic exploration confirmed a 4 cm × 1.5 cm transverse full-thickness rupture on the anterior uterine wall, 2 cm inferior to the round ligament insertion (Figure 2F). Consequently, the procedure was converted to an open laparotomy. Active bleeding was also observed at the uterine fundus, where the surface appeared irregular. The myometrial tissue surrounding the uterine rupture exhibited poor elasticity. The ruptured area was trimmed, followed by continuous full-thickness suturing of the uterine muscle layer. Subsequently, the superficial myometrium was closed over the rupture site using vertical mattress sutures to embed the wound. Approximately 800 mL of blood clots and a total of around 2000 mL of blood were evacuated from the pelvic and abdominal cavities. One drainage tube was placed in the pelvic cavity. In conclusion, the progressive anemia and corroborative imaging findings justified diagnostic laparoscopy, which ultimately revealed significant hemoperitoneum and a full-thickness uterine rupture.

### 2.4 Postoperative stay and follow-up

During the intraoperative procedure, a female fetus (430 g) was delivered and promptly transferred to the pediatric intensive care unit for emergency treatment. The uterine defect was subsequently repaired in two layers. The patient underwent blood product transfusion, was transferred to the intensive care unit postoperatively, and was discharged on postoperative day 5 without complications. At the three-month postoperative follow-up, the patient recovered well, with no clinical or imaging evidence of uterine pathology.

## 3 Discussion

Uterine rupture is a rare but life-threatening obstetric complication that predominantly occurs during late pregnancy or the active phase of labor. Its clinical manifestations are highly variable, depending on the rupture site, morphological characteristics, degree of vascular injury, and placental implantation status, with symptoms ranging from subclinical presentations to hemorrhagic shock (2–4). This variability often leads to misdiagnosis. Classic symptoms include lower abdominal tenderness and abnormal fetal heart rate patterns. Timely diagnosis and treatment are crucial for improving maternal and fetal outcomes (5–7).

We report the case of a multiparous woman at 22 weeks and 3 days of gestation who presented with acute upper abdominal pain radiating to the shoulder and back. She had a history of HIFU ablation for uterine fibroids and prior uterine instrumentation. Imaging revealed multifocal intraperitoneal hemorrhage, which complicated the diagnostic process. This case provides valuable clinical insights into the evaluation of acute abdominal pain during pregnancy.

The primary risk factors for uterine rupture in the second trimester include cesarean section and myomectomy-induced scarred uterus, uterine malformations, history of intrauterine procedures, advanced maternal age, and multiple pregnancies, with scarred uterus being the most critical (8–10).

Scarless uterine rupture typically manifests as sudden severe abdominal pain, abnormal fetal heart rate, and intra-abdominal hemorrhage. In contrast, uterine rupture following myomectomy may present with atypical symptoms, with some cases exhibiting “silent rupture” characterized by hemodynamic stability and a normal fetal heart rate (11). The present case involves a scarred uterus treated with HIFU, and its clinical presentation closely aligns with previously reported features of uterine rupture with hemodynamic stability and a normal fetal heart rate.

Case analysis of uterine rupture after HIFU treatment indicates that this complication primarily occurs during the middle and late stages of pregnancy (12). The pathogenesis is closely associated with abnormal tissue repair or poor scar formation in the treatment area, with specific pathophysiological mechanisms including coagulative necrosis in target tissues and apoptosis of surrounding cells. Abnormal scar repair and hemodynamic changes collectively reduce the load-bearing capacity of the uterine wall (13, 14).

In this case, the patient experienced uterine rupture 3 years after HIFU therapy, in the setting of a scarred uterus complicated by poorly controlled gestational diabetes mellitus, which likely exacerbated uterine structural weakness and ultimately led to mid-trimester rupture. Therefore, obstetricians and emergency physicians should recognize that both prior HIFU therapy and diabetes mellitus are potential risk factors for uterine rupture, warranting heightened clinical vigilance.

Guidelines for laparoscopic surgery in non-obstetric emergencies during pregnancy recommend that appendectomy and biliary tract diseases should prioritize laparoscopic (15). In this context, the application of emergency laparoscopic exploration in cases of visceral rupture with unidentified bleeding sites demonstrates exceptional diagnostic accuracy and enables precise surgical intervention. The patient exhibited high-density fluid accumulation in the perihepatic, perisplenic, and perienteric regions, accompanied by progressive hemoglobin decline and hemodynamic instability, consistent with the clinical presentation of hemorrhagic shock. In this instance, prompt multidisciplinary consultation enabled successful laparoscopic localization of the hemorrhage. It reduces laparotomy exploration time and serving as a pivotal determinant of therapeutic success.

## 4 Conclusion

Uterine rupture can present with predominant epigastric pain and may be occult on advanced imaging. Clinicians should maintain vigilance for obstetric emergencies in any pregnant patient with acute abdominal pain, especially when risk factors such as prior uterine surgery exist. Prompt multidisciplinary evaluation, routine collaboration between specialized hospitals and general hospitals, and early surgical exploration are essential to improve maternal outcomes.

## Data Availability

The original contributions presented in the study are included in the article/supplementary material, further inquiries can be directed to the corresponding authors.
